# A deep learning approach to automatic gingivitis screening based on classification and localization in RGB photos

**DOI:** 10.1038/s41598-021-96091-3

**Published:** 2021-08-19

**Authors:** Wen Li, Yuan Liang, Xuan Zhang, Chao Liu, Lei He, Leiying Miao, Weibin Sun

**Affiliations:** 1 Department of Endodontics, Nanjing Stomatological Hospital, Medical School of Nanjing University, No.30 Zhongyang Road, Xuanwu District, Nanjing, Jiangsu People’s Republic of China; 2 University of California, Los Angeles, USA; 3 Department of Periodontics, Nanjing Stomatological Hospital, Medical School of Nanjing University, No.30 Zhongyang Road, Xuanwu District, Nanjing, Jiangsu People’s Republic of China; 4 Department of Orthodontics, Nanjing Stomatological Hospital, Medical School of Nanjing University, Nanjing, People’s Republic of China

**Keywords:** Diseases, Health care, Medical research

## Abstract

Routine dental visit is the most common approach to detect the gingivitis. However, such diagnosis can sometimes be unavailable due to the limited medical resources in certain areas and costly for low-income populations. This study proposes to screen the existence of gingivitis and its irritants, i.e., dental calculus and soft deposits, from oral photos with a novel Multi-Task Learning convolutional neural network (CNN) model. The study can be meaningful for promoting the public dental health, since it sheds light on a cost-effective and ubiquitous solution for the early detection of dental issues. With 625 patients included in this study, the classification Area Under the Curve (AUC) for detecting gingivitis, dental calculus and soft deposits were 87.11%, 80.11%, and 78.57%, respectively; Meanwhile, according to our experiments, the model can also localize the three types of findings on oral photos with moderate accuracy, which enables the model to explain the screen results. By comparing to general-purpose CNNs, we showed our model significantly outperformed on both classification and localization tasks, which indicates the effectiveness of Multi-Task Learning on dental disease detection. In all, the study shows the potential of deep learning for enabling the screening of dental diseases among large populations.

## Introduction

Gingivitis is the chronic infection of oral gum that has been affecting the public health worldwide, whose main manifestations are redness, bleeding, and bad breath. The latest epidemiological survey on oral health showed that 87.4% of adults aged 35–44 years suffers from the resultant gingival bleeding^1^. The development of gingivitis is a continuous progress, with dental calculus and soft mucinous deposits being the main irritants^2^. Soft deposits is a visible bacterial mass on teeth, while dental calculus is a mineralized soft deposit that further destroys the periodontal tissues by constantly adsorbing calcium compounds from saliva. The awareness of gingivitis and its irritants can be helpful for individuals to stall and control the progress of gingivitis with corresponding interventions, as well as getting timely treatment to avoid tooth loss. Currently, routine dental visit is the most effective way for such detection. However, the dental diagnosis is not always available due to the limited medical resources in some regions. Also, it can add economic burdens for individuals of low-income, which might prevent them from routine dental visits^3^. Therefore, there has been a need for cost-effective solutions that can screen for gingivitis and its irritants, i.e., dental calculus and soft deposits, among large populations. Motivated by the need, this study sheds light on the development of computer-assisted systems that utilizes widely available oral photos for the purpose of dental health screening.

In specific, ur work employs deep learning (DL) algorithms for highly efficient and accurate disease detection^4^. Currently, DL has been widely used to detect health issues from imagery captured with cameras or smartphones for everyday users as a supplement to clinical visits^5,6^. Indeed, BiliScreen achieved the automatic live disorder detection by capturing Jaundice color changes^7^; Vardell introduced a skin disease diagnosis system with skin photos as a source of input^8^; and Mariakakis et al. developed the fast traumatic brain injury detection by training a model taken pupil images as input^9^. Despite those works, the use of DL models for screening oral conditions is still much under-explored.

There also exists continuous efforts for enabling the automatic dental diagnosis with DL algorithms. For example, Joachim Krois^10^ applied CNNs to detect periodontal bone loss (PBL) on panoramic dental radiographs, while Casalegno et al. performed caries segmentation from Near-Infrared-Light Transillumination (NILT) images^11^. Jae-Hong Lee evaluate the efficacy of deep CNN algorithms for detection and diagnosis of dental caries on periapical radiographs^12^. Yu et al. also evaluated the performance of CNNs for the skeletal classification with lateral cephalometry^13^. Different from the previous work, our task takes imageries as input, which can be less standard in distribution comparing to the medical imaging.

In this work, we initialize the study of applying DL on oral photos for screening gingivitis, dental calculus, and soft deposits. We formulated the task as mixture of dental condition classification and localization by considering the nature of the conditions, and developed a Multi-Task Learning to solve the two different types of tasks with an integrated model. We proved the effectiveness of our model by comparing with general-purpose CNNs and carrying out ablation tests. With the designed system, we expect to bring up the discussion for integrating deep learning into tools for improving public dental health.

## Materials and methods

In this section, we first introduce the data collecting protocols and the resultant dataset for the data-driven study, followed by the description of data annotation process. Next, we illustrate the problem formulation for the detection of gingivitis (and its irritants), as well as the proposed DL model architecture. Details on the model implementation and training are then provided. Finally, we describe the metrics and statistical analysis methods we applied for validating and justifying our model.

### Acquisition of data

A total of 3932 oral photos were captured from 625 patients admitted at Department of Periodontics, orthodontics and endodontics, Nanjing Stomatological Hospital, Nanjing University, between January 2018 and December 2019. All the photos were captured by postgraduate dental students and dentists, and the patient’s ages cover a range from 14 to 60. The project was approved by the Ethical Review Board at Nanjing University (approval 2019NL-065(KS)). The methods were conducted in accordance with the approved guidelines, written informed consent was obtained from each participant. For children under the age of 18, the written informed consent was obtained from their parents/guardians. To approximate the image quality in the practical scenario, the photos were collected with various equipments which include iPhone 8, iPhone 7, Samsung Galaxy s8, and Canon 6D. No specific in- or exclusion criteria about images, e.g. lighting and resolution, was applied. Three dental diseases were considered in this study: gingivitis, dental calculus, and soft deposit. Among the dataset, 3175 photos show gingivitis, 921 show dental calculus, and 746 images show soft deposits. Note that each photo can show none, one or more types of conditions. All photos were pseudonymized and no other image processing steps are performed.

We split the data into training, validation and testing subsets by randomly splitting the patients into three independent groups. All photos of each patient only existed in one of the three subsets. Table 1 shows the patient and photo split. Table 2 shows the distribution of photos with positive findings among the dataset and different subsets.

**Table 1:** Numbers of patients and images assigned to training, validation, and testing subsets.

	Training	Validation	Testing	Total
Patients	344 (55.04%)	94 (15.04%)	187 (29.92)	625 (100%)
Images	2138 (54.37%)	608 (15.46%)	1186 (30.16%)	3932 (100%)

**Table 2:** Distribution of images with positive findings among training, validation, and testing subsets for each type of diagnosis.

	Gingivitis	Dental calculus	Soft deposit
Training	1726 (80.73%)	514 (24.04%)	424 (19.83)
Validation	469 (77.14%)	175 (28.78%)	116 (19.08%)
Testing	980 (82.63%)	232 (19.56%)	206 (17.37%)
Total	3175 (80.75%)	921 (23.42%)	746 (18.97%)

The data shows the numbers of images with positive findings, as well as their proportions within all the images of a category.

### Ground truth annotations

We collected the reference annotations of the three dental conditions for all the photos from three board-certified dentists. In specific, the dataset was evenly split and assigned to the three dentists. Each image was independently labeled by one of the dentists with the given clinical report using the labeling software LabelBox (Labelbox, Inc, CA). For gingivitis and dental calculus, we collected the annotations of bounding boxes for indicating the localizations of diseases. Note that since there can be no well-defined boundaries for diseases in some cases, we followed a common approach^14^ by instructing the dentists to focus on the correctness of box centers. Meanwhile, for soft deposit, we only collected image-level classification labels, since such condition mostly appears all over cavities and its localizations are labor-costly to label.

### Problem formulation and model architecture

**Figure 1:**
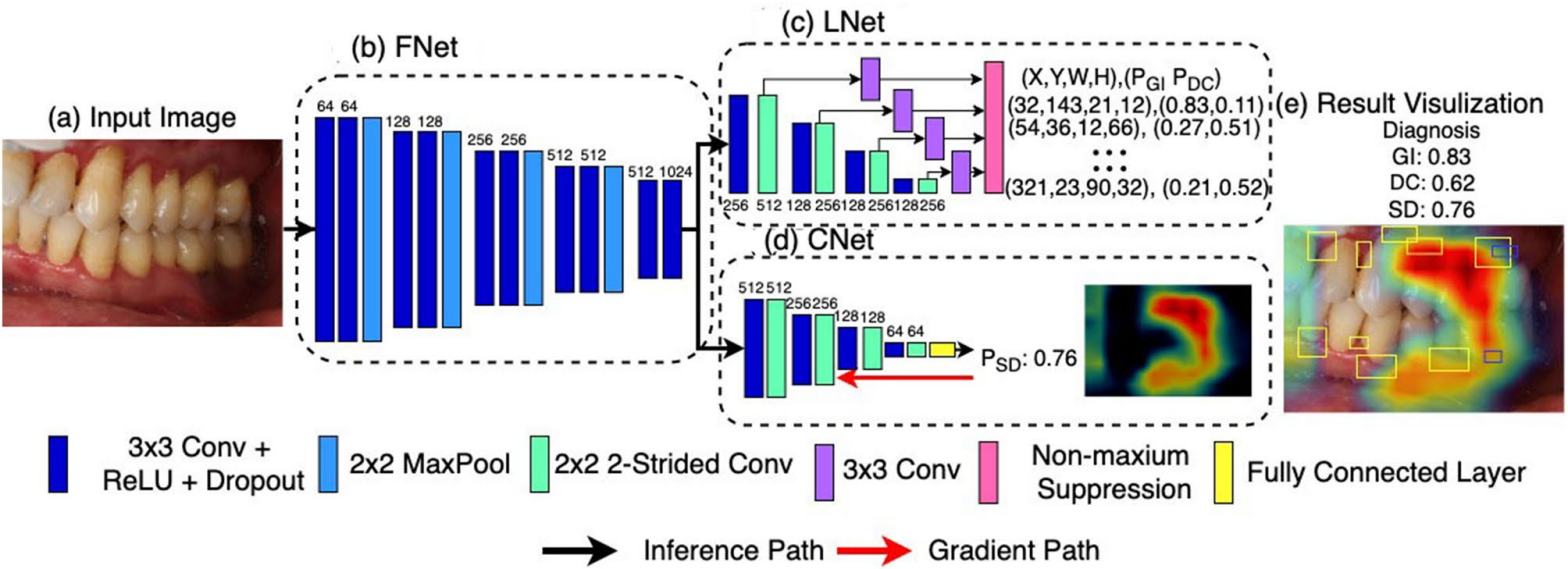
Overview of the model architecture. The model consists of three subnets: (**b**) FNet of feature extraction, (**c**) LNet for bounding box-based localization, and (**d**) CNet for classifying the existence of conditions. Given an (**a**) oral cavity image as input, (**e**) the model outputs both the probabilities for diagnosis, as well as the locations of the detections with heat-maps and bounding boxes. The numbers at model building blocks mark for channel dimensions. Gingivitis, dental calculus, and soft deposits are represented as GI, CA, and SD, respectively.

We formulated the problem as a mixture of object localization and image classification. In specific, we developed a CNN with Multi-task Learning (MTL)^15,16^ for solving both tasks with a unified model in order to increase the model’s generalization^17^ and compactness. Figure 1 shows the overall architecture of our MTL model, which takes oral images (Fig. 1a) as input, and outputs both diagnosis and locations of the detected conditions (Fig. 1e). The model is consisted of three subnets: (i) FNet (feature extraction subnet), (ii) LNet (localization subnet) and (iii) CNet (classification subnet). FNet (Fig. 1b) extracts deep features of the input image through a stack of convolutional layers, and was trained to be discriminative for both localization and classification tasks. Meanwhile, LNet (Fig. 1c) regresses over the feature maps derived from FNet for a set of location vectors, where each vector y encodes one bounding box by its coordinates, height, width, and probability for gingivitis and dental calculus. Similar with Liu et al.^18^, the proposed bounding boxes are aligned to nearest ground-truth boxes during training to approximate localizations, and are filtered with Non-maximum Suppression (NMS)^19^ during inference to reduce overlapped findings. CNet (Fig. 1d) performs fully connected operations over the extracted feature maps for a length 1 vector as outputs, whose value represented the probability for the existence of soft deposits. By optimizing the whole model consisted of FNet, CNet and LNet end-to-end, we enforce the FNet to learn representations that are effective for both classification and localization. Such constraint can possibly improve the generalization of the representations and reduce the model overfitting.

To help users comprehensively understand the diagnosis results, we aim to highlight the spatial locations of the detected dental conditions. For gingivitis and dental calculus, the bounding boxes from the model can already localize the ROIs. However, for soft deposit, the model only produces classification results since their ground-truth location maps are labor costly to annotate. Thus, gradient-based class activation maps^20^ was used to reason the areas of the images that are most indicative to the classification.

Figure 1e (Fig. 1 were created with Matplotlib v3.2.1 (https://matplotlib.org/) shows an example result from our system. The detected gingivitis and dental calculus are pinpointed with boxes, and soft deposits are hinted with a heat-map, where a higher temperature indicates the stronger relevance of a region. The whole model can be optimized end-to-end during training, and can produce both the classification and localization results in a single run during inference.

### Implementation and training strategy

To train the model, we defined the loss as an equally weighted sum of smooth L1 loss for bounding box regression, and cross entropy loss for classification^21^. We employed intensive augmentations to input images^22^, which includes random shifts, crops, rotations, scaling, and color channel shifts (random changes of hue, saturation, and exposure). Such augmentations is targeted to increase the robustness of the model for in-the-wild application. Moreover, we employed transfer learning by initializing our FeatNet from VGG-16^23^ that pre-trained on large-scale image recognition tasks for speeding up the training process^24^.

The CNN model was developed using the PyTorch framework. The model was trained using a mini batch size of 16 per GPU on three Nvidia 1080 Ti GPUs. Validation set was used to determine the early stopping of the training process. Parameter updates were calculated using the Adam algorithm, with the learning rate set to 1e−4 and decay rate set to 5e−4.

### Evaluation metrics and statistical analysis

We evaluated the model from two aspects: (i) classification performance for telling the existence of a condition, and (ii) localization performance for indicating regions on images that related to a diagnosis.

In terms of the classification performance, we utilized the Receiver Operating Characteristic (ROC) curve, which shows the true-positive rate (TPR), or sensitivity, against its false-positive rate (FPR), or 1 − specificity, as a function of varying discrimination thresholds. The ROC curve illustrates the diagnostic ability of binary classifier. For gingivitis and dental calculus, we took the highest probability of the detected bounding boxes as the classification probability of an image; meanwhile for soft deposit the classification model output is taken as the probability. To compare between different models, Area Under the Curve (AUC) was used as a numeric measurement of class separability, where a higher value indicates the better model performance.

In terms of localization performance, we utilized the Free-Response ROC (FROC) curve, a commonly used graphic measurement for medical anomaly detection^25–29^. In the FROC paradigm, a model is free to mark as many clinically suspicious regions; a mark is true positive if it is sufficiently close to an actual anomaly, otherwise it is scrod a a location-wise false positive. FROC measures the location-wise TPR against the average number of false-positive (FP) locations per image as a function of varying thresholds for box probabilities. Moreover, by following the practice of van Ginneken^24^, a predicted box was taken as a hit if its center falls into the range of a ground-truth box. To conveniently compare different models numerically, we followed Setio^28^ and van Ginneken^24^ to define a Localization Performance Metric (LPM) as the average sensitivity at the false positive numbers per image of 1/2, 1, 2, and 3.

In terms of measuring the quality of soft deposit localization, we followed Selvaraju^20^ by collecting agreement ratings from three board-certified dentists for each localization heat-map of testing images. Specifically, we show dentists images that were detected with soft deposits together with the localization heat-maps that visualized as in Fig. 1e. Then a rating is given on a scale from 1 (strongly disagree) to 5 (strongly agree) by evaluating if a heat-map demonstrates the regions of the condition according to dentists’ opinions.

## Results

Tables 3 and 4 show the classification and localization performance of different models, respectively. Compared to the general-purpose classification CNNs (VGG-1621 and Residual-5027) and localization CNNs (SSD 20), our model has the advantage of handling both types of tasks. The model achieved classification AUC (95% CI) of 87.11 (82.27 to 91.49) for gingivitis, 80.11% (CI 75.99% to 84.45%) for dental calculus, and 78.57% (CI 74.32% to 82.78%) for soft deposits; meanwhile the model performed at LPM (95% CI) of 58.19% (56.15% to 60.20%) and 49.39% (44.40% to 54.69%) for localizing gingivitis and dental calculus, respectively. All the scores were highest scores among different methods, suggesting the effectiveness of the proposed system. Additionally, we conducted an ablation test comparing the performance of our model (FeatNet + ClassNet + LocateNet) with its subnets that trained solely for classification (FeatureNet + classNet) or localization (FeatureNet + locateNet). The results show that our model outperformed in all metrics, with AUC boosts ranging from 1.20 to 8.82%, and LPM boosts ranging from 1.26 to 5.52%. The results confirm the advantage of handling multiple tasks simultaneously with joint optimization.

**Table 3:** Summary of classification performance of different models. classification AUC (in percentage), sensitivity (Sens.), specificity (Specif.) are measured for gingivitis (GI), dental calculus (DC), and soft deposits (SD).

	GI AUC (95% CI)/%	GI sens.	GI specif.	DC AUC (95% CI)/%	DC sens.	DC specif.	SD AUC (95% CI)/%	SD sens.	SD specif.
VGG-16	77.55 (73.97 to 79.05)	0.682	0.620	74.17 (72.71 to 76.75)	0.645	0.572	72.07 (69.76 to 76.34)	0.679	0.605
Residual-50	83.80 (80.69 to 86.37)	0.755	0.628	78.22 (75.91 to 82.02)	0.780	0.567	71.49 (67.86 to 75.98)	0.641	0.586
FNet + CNet	84.28 (80.94 to 87.11)	0.802	0.578	76.78 (74.50 to 79.47)	0.745	0.615	69.75 (68.29 to 71.57)	0.740	0.480
Ours (high-sensitivity operation point)	87.11 (82.27 to 91.49)	0.878	0.639	80.11 (75.99 to 84.45)	0.778	0.655	78.57 (74.32 to 82.78)	0.787	0.590
Ours (high- specificity operation point)	87.11 (82.27 to 91.49)	0.601	0.839	80.11 (75.99 to 84.45)	0.542	0.836	78.57 (74.32 to 82.78)	0.565	0.800

**Table 4:** Summary of localization performance of different models. detection FAUC (in percentage), bounding-box-wise sensitivity (Sens.), average false positives (Avg. False Positives) are measured for gingivitis (GI) and dental calculus (DC).

	GI FAUC (95% CI)/%	GI sens.	GI avg. false positives	DC FAUC (95% CI)/%	DC sens.	DC avg. false positives
SSD	44.19 (41.19 to 47.08)	0.422	1.280	36.23 (32.93 to 37.89)	0.375	1.721
FNet + LNet	56.93 (56.05 to 59.42)	0.565	1.340	43.87 (41.26 to 48.33)	0.392	1.150
Ours (high-sensitivity operation point)	58.19 (56.15 to 60.20)	0.666	1.522	49.39 (44.40 to 54.69)	0.456	1.330
Ours (high-specificity operation point)	58.19 (56.15 to 60.20)	0.432	0.585	49.39 (44.40 to 54.69)	0.380	0.260

**Figure 2:**
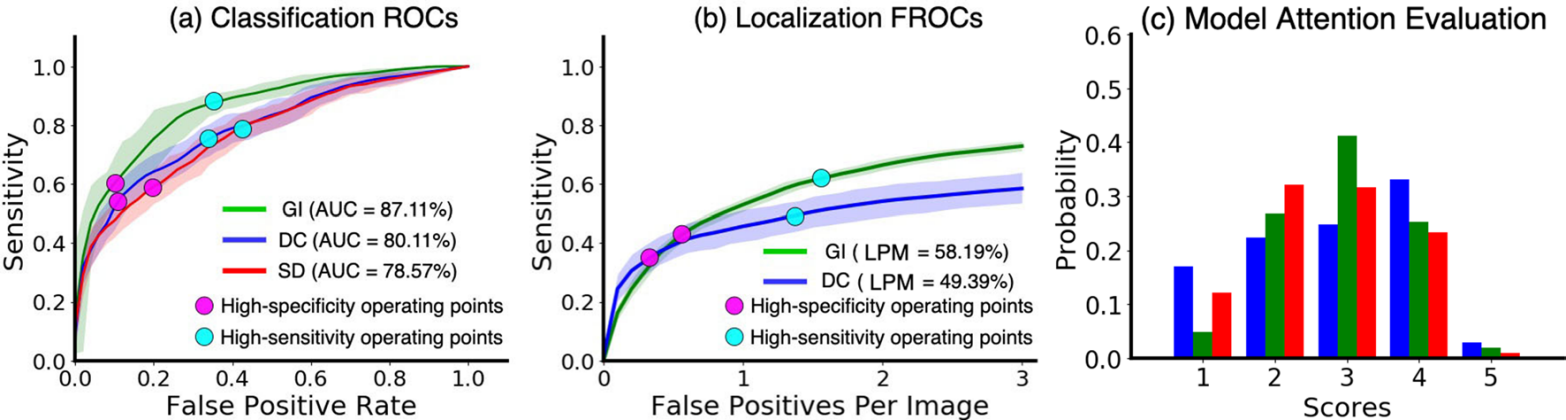
Quantitative analysis of the results of our model. (**a**) Receiver operating characteristic curves for predicting the existence of conditions. (**b**) Free-Response receiver operating characteristic curves for localizing conditions. (**c**) Distribution of dentists’ ratings on the localization for soft deposits. Gingivitis, dental calculus, and soft deposits are represented as GI, DC, and SD, Respectively.

The ROC and FROC curves for each diagnosis are detailed in Fig. 2a,b. The designed operating points of the model are shown with black diamonds and red dots. Two types of operating points were designed by following^30,31^: the high-specificity operating point with a higher discrimination threshold that aims for reducing false positives, and the high-sensitivity operating point with a lower discrimination threshold for keeping the missing rate low. The model achieved the mean specificities of 83.87%, 83.61%, and 79.98% under the high-specificity operating points, meanwhile the mean sensitivities of 87.83%, 77.79%, and 78.68% under the high-sensitivity operating points, both for gingivitis, dental calculus, and soft deposit, respectively. For localization performance, the model achieved the mean box-wise sensitivities of 66.57% and 45.61% for gingivitis and dental calculus, respectively, at the high-sensitivity operating point. Moreover, Fig. 2c shows the dentists’ ratings on the attention-based localization for soft deposits. While the attention-based method has been widely applied to interpret CNNs^32,33^, it lacks formal evaluations of location-indicating accuracy for the dental diagnosis purpose. According to the experiment, our model achieved scores with a median of 3.00, mean of 2.81, and standard deviation of 1.02, on a scale from 1 to 5. Based on the scorers’ feedback, the following two factors can lead to the lower localization scores. First, different from bounding boxes that pinpointing the exact locations, the heat-maps can only circle out areas with larger ranges. Second, the model attention can often localize only part of the related regions. This can be explained as the model does not count on all regions for reaching a classification results^20,32^.

**Figure 3:**
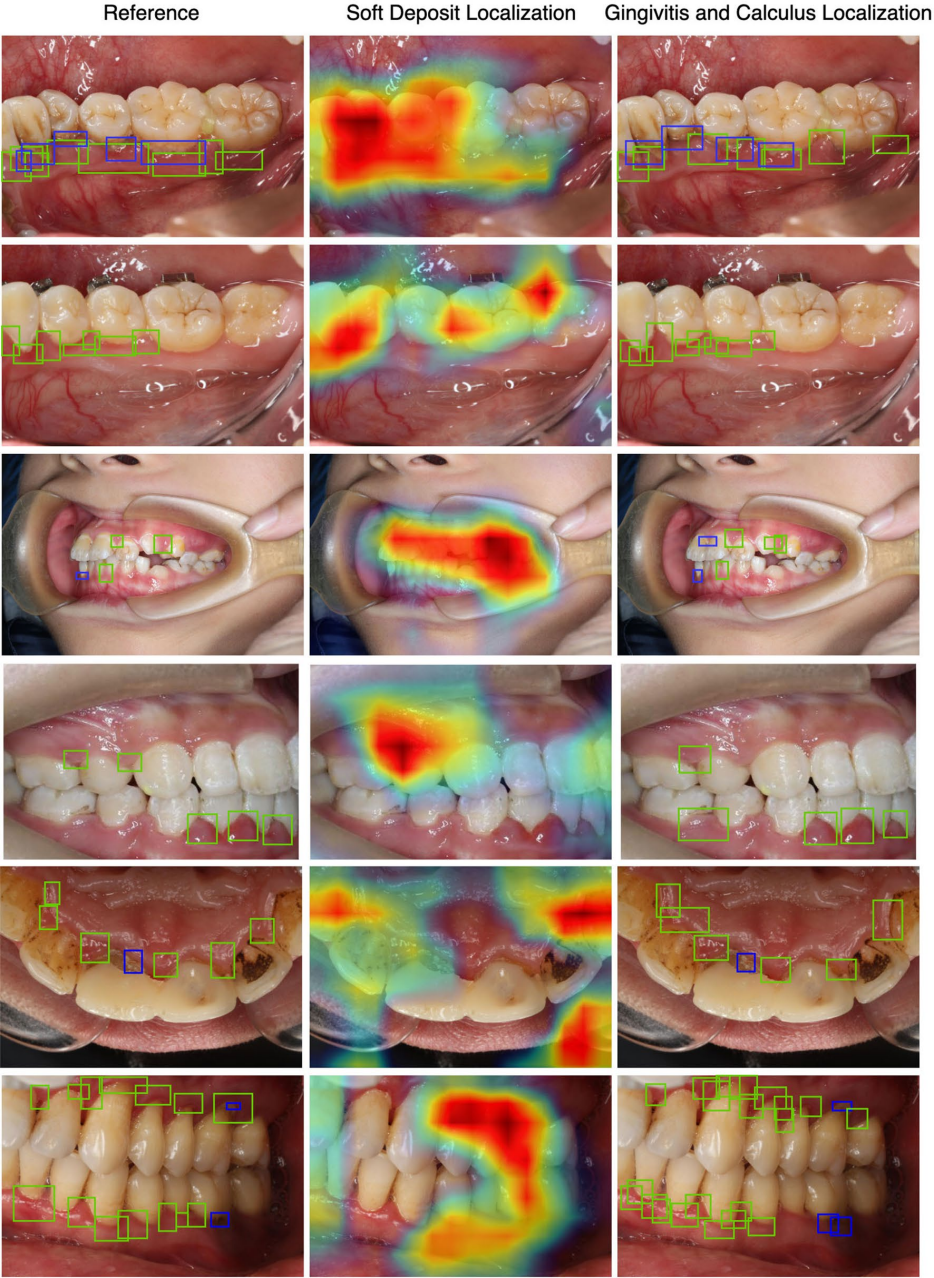
Examples of detection results on the testing data. Left: ground-truth annotations. Middle: Predicted locations of soft deposits represented with heat-map. Right: predicted locations of gingivitis and dental calculus represented with green and blue boxes, respectively.

Figure 3 (Fig. 3 were created with Matplotlib v3.2.1 (https://matplotlib.org/).depicts selected results obtained on the testing images for a qualitative overview of our model’s performance. Ground-truth annotations, heat-map predictions and bounding box predictions are shown in the left, middle, and right column. We can clearly see that the model can accurately tell the existence of dental conditions with acceptable accuracy of localization. By looking into the outputs, we found that the over-exposure, under-exposure and incorrect focus of photos can lead to wrong predictions.

## Discussion and future work

Previous studies have explored predicting gum health with self-reported questionnaires^34,35^. Their results have shown that several self-reported measures and risk factors are strongly related to the presence of gum diseases. Different from those works, we aim to predict gingivitis as well as its irritants as early indicators from oral photos by learning their common appearance patterns. Such visual signals can be of more direct reflection of dental diseases than questionnaire feedbacks. Moreover, the method is promising since oral photos can be collected with smartphones, which have become increasingly low-cost and ubiquitous recently. Our work pioneers to examine the approach by designing, training and validating a deep learning model for the task. Built based on the detection results of deep learning, future systems can be developed to show targeted health-enhancing activities, proper hygiene routines, and clinical treatments to users, which will be meaningful for promoting the public dental health.

Considering that the users of such system can have limited knowledge about dental health, our model shows not only the existences of dental conditions but also their localizations. The localization can help users better understand the screening results, and help gain trust of users to a system with the increased explainability^36^. We formulated the localization of gingivitis and dental calculus as bounding box regression by considering the appearance of the conditions and saving labour cost. For soft deposits, we formulated the task as image-wise classification, and reasoned its locations with model attentions.

To improve the system efficiency, we employed Multi-Task Learning, such that both types of tasks, i.e. classification and localization, can be solved with one integrated CNN model. Moreover, our experiments indicated that our model also outperformed the state-of-the-art CNNs that carried out single type of task in accuracy, mainly because the co-optimization of multiple tasks increases the model generalization. We further confirmed this with ablation tests, where the model with MTL showed significant accuracy improvements comparing to its subnets that trained for classification or localization solely. We believe the findings can help with the CNN design for other dental diagnosis with multiple goals.

Our work still exhibits several shortcomings, and we discuss the possible solutions for future researches. First, our dataset is limited in the sense that the photos were collected from a single organization, and currently it only covered age range from 14 to 60. We have the plan to further enrich the dataset for a wider age range from multiple sites globally. Second, our model achieved a relative low accuracy on soft deposit for the localization task, partially due to the lack of spatial annotations as the guidance for training supervision. Instead of collecting pixel-wise segmentation maps, which can be extremely labour costly, we advocate that future studies could apply recently proposed weakly-supervised learning to train with low-quality spatial annotations, e.g. partial labels over images to indicate several typical areas of diseases^37^. Moreover, the model could also benefit from semi-supervised learning by augmenting a part of dataset with pixel-wise labels^38,39^, while the other part only comes with image-wise labels. Third, the algorithm can also be complementary with the traditional questionnaire-based detections for higher reliability and accuracy. The current model cannot utilize data modality other than images for diagnosis. Encoding^40^ and fusing of medical history and self-reported symptoms of a patient into CNNs could be promising to improve the model accuracy^41^.

## Conclusion

In this study, a deep learning model for the detection of gingivitis, dental calculus, and soft deposits from oral photos was proposed. We formulated the model with Multi-Task Learning, which effectively improves its compactness and accuracy. We evaluated our model for both classification and localization tasks. Based on the results, we show deep learning is promising for enabling the cost-effective screening of dental diseases among large populations from oral photos, which can captured with smartphones and other commonly available devices. Built upon the deep learning model, systems can be developed to provide user-specific health-enhancing activities according to one’s dental conditions, which can be promising to improve the public dental health. Our work also discusses the possible improvements of data quantity and model architectures.

## Data Availability

The data used in current study were collected from Medical School of Nanjing University and is available only for the granted research. However, the data can be made available if requested within data protection and regulation guideline.

## References

[1] Supranoto SC, Slot DE, Addy M, Ga VDW (2015). The effect of chlorhexidine dentifrice or gel versus chlorhexidine mouthwash on plaque, gingivitis, bleeding and tooth discoloration: A systematic review. Int. J. Dental Hygiene.

[2] Miyauchi S (2017). Sphingomyelin phosphodiesterase 3 enhances cytodifferentiation of periodontal ligament cells. J. Dent. Res..

[3] Petersen PE, Bourgeois D, Ogawa H, Estupinan-Day S, Ndiaye C (2005). The global burden of oral diseases and risks to oral health. Bull. World Health Organ..

[4] Liu YP, Li Z, Xu C, Li J, Liang R (2019). Referable diabetic retinopathy identification from eye fundus images with weighted path for convolutional neural network. Artif. Intell. Med..

[5] Litjens G (2017). A survey on deep learning in medical image analysis. Med. Image Anal..

[6] Wang G (2018). Interactive medical image segmentation using deep learning with image-specific fine tuning. IEEE Trans. Med. Imaging..

[7] Mariakakis A (2017). Biliscreen: Smartphone-based scleral jaundice monitoring for liver and pancreatic disorders. Proc. ACM Interact. Mobile Wearable Ubiquitous Technol..

[8] Vardell E, Bou-Crick C (2012). VisualDx: A visual diagnostic decision support tool. Med. Ref. Serv..

[9] Mariakakis A (2017). PupilScreen: Using smartphones to assess traumatic brain injury. Proc. ACM Interact. Mobile Wearable Ubiquitous Technol..

[10] Krois J (2019). Deep learning for the radiographic detection of periodontal bone loss. Sci. Rep..

[11] Casalegno F (2019). Caries detection with near-infrared transillumination using deep learning. J. Dent. Res..

[12] Lee JH, Kim DH, Jeong SN, Choi SH (2018). Diagnosis and prediction of periodontally compromised teeth using a deep learning-based convolutional neural network algorithm. J. Periodontal Implant Sci..

[13] Yu HJ (2020). Automated skeletal classification with lateral cephalometry based on artificial intelligence. J. Dent. Res..

[14] Armato SG (2007). The lung image database consortium (LIDC): Ensuring the integrity of expert-defined "truth"1. Acad. Radiol..

[15] Ranjan, R., Sankar, S., Castillo, C. D. & Chellappa, R. An all-in-one convolutional neural network for face analysis. In *2017 IEEE International Conference on Automatic Face & Gesture Recognition (FG 2017)*, pp. 17–24 (IEEE, 2017).

[16] Bansal, A., Nanduri, A., Castillo, C., Ranjan, R. & Chellappa, R. Umdfaces: An annotated face dataset for training deep networks. In *2016 IEEE International Joint Conference on Biometrics (IJCB),* (IEEE, 2016).

[17] Krizhevsky A, Sutskever I, Hinton GE (2012). Imagenet classification with deep convolutional neural networks. Adv. Neural. Inf. Process. Syst..

[18] Liu, W., Anguelov, D., Erhan, D., Szegedy, C. & Berg, A. C. SSD: Single shot multibox detector. In *European Conference on Computer Vision*, pp. 21–37 (Springer, 2016).

[19] Simonyan, K. & Zisserman, A. Very deep convolutional networks for large scale image recognition. *Computer Science*. *arXiv preprint *arXiv:1409.1556 (2014).

[20] Selvaraju, R.R. *et al.* Grad-cam: Visual explanations from deep networks via gradient-based localization. In *Proceedings of the IEEE International Conference on Computer Vision*, pp. 42–48 (IEEE, 2017).

[21] Esteva A (2017). Dermatologist-level classification of skin cancer with deep neural networks. Nature.

[22] Redmon, J. & Farhadi, A. YOLO9000: better, faster, stronger. In *Proceedings of the IEEE Conference on Computer Vision and Pattern Recognition*, pp. 7263–7271 (IEEE, 2017).

[23] Deng, J. *et al.* Imagenet: a large scalehierarchical image database. In *2009 IEEE Conference on Computer Vision and Pattern Recognition*, pp. 643–651 (IEEE, 2009).

[24] van Ginneken B (2010). Comparing and combining algorithms for computer-aided detection of pulmonary nodules in computed tomography scans: The ANODE09 study. Med. Image Anal..

[25] Chartrand G, Cheng PM, Vorontsov E, Drozdzal M, Turcotte S, Pal CJ, Kadoury S, Tang A (2017). Deep learning: A primer for radiologists. Radiographics.

[26] Wang Z, Liu C, Cheng D, Wang L, Yang X, Cheng KT (2018). Automated detection of clinically significant prostate cancer in mp-MRI images based on an end-to-end deep neural network. IEEE Trans. Med. Imaging.

[27] Chakraborty DP (2013). A brief history of free-response receiver operating characteristic paradigm data analysis. Acad. Radiol..

[28] Setio AA, Traverso A, De Bel T, Berens MS, Van Den Bogaard C, Cerello P, Chen H, Dou Q, Fantacci ME, Geurts B, van der Gugten R (2017). Validation, comparison, and combination of algorithms for automatic detection of pulmonary nodules in computed tomography images: The LUNA16 challenge. Med. Image Anal..

[29] Yan K, Wang X, Lu L, Summers RM (2018). DeepLesion: Automated mining of large-scale lesion annotations and universal lesion detection with deep learning. J. Med. Imaging.

[30] Gulshan V (2016). Development and validation of a deep learning algorithm for detection of diabetic retinopathy in retinal fundus photographs. JAMA.

[31] De Vrijer M, Medendorp WP, Van Gisbergen JAM (2009). Accuracy-precision trade-off in visual orientation constancy. J. Vis..

[32] Montavon G, Samek W, Müller KR (2018). Methods for interpreting and understanding deep neural networks. Digit. Signal Process..

[33] Jalali A (2020). Deep learning for improved risk prediction in surgical outcomes. Sci Rep..

[34] Beltrán-Aguilar ED, Eke PI, Thornton-Evans G, Petersen PE (2012). Recording and surveillance systems for periodontal diseases. Periodontol..

[35] Eke PI (2013). Self-reported measures for surveillance of periodontitis. J. Dent. Res..

[36] Gilpin LH (2018). Explaining Explanations: An Overview of Interpretability of Machine Learning.

[37] Khoreva, A., Benenson, R., Hosang, J., Hein, M. & Schiele, B. Simple does it: Weakly supervised instance and semantic segmentation. In *Proceedings of the IEEE Conference on Computer Vision and Pattern Recognition*, pp. 825–833 (IEEE, 2016).

[38] Bai, W. *et al.* Semi-supervised learning for network-based cardiac MR image segmentation. In *International Conference on Medical Image Computing and Computer-Assisted Intervention*, pp. 253–260 (Springer, 2017).

[39] Cheplygina V, de Bruijne M, Pluim JPW (2019). Not-so-supervised: A survey of semi-supervised, multi-instance, and transfer learning in medical image analysis. Med. Image Anal..

[40] Choi E, Xiao C, Stewart WF, Sun J (2018). Mime: Multilevel medical embedding of electronic health records for predictive healthcare. Adv. Neural. Inf. Process. Syst..

[41] Esteva A (2019). A guide to deep learning in healthcare. Nat. Med..

